# Hepatitis B Virus Vaccination Coverage, Compliance, and Barriers Among Healthcare Workers in Lemi Kura Subcity, Addis Ababa, Ethiopia: A Mixed‐Approach Study

**DOI:** 10.1155/bmri/9163340

**Published:** 2026-04-02

**Authors:** Samuel Dessu Sifer, Nebyu Lakew Tefera, Abenezer Zenebe Kebede, Melkamu Abte, Mulugeta Geremew

**Affiliations:** ^1^ Department of Epidemiology and Biostatistics, School of Public Health, Yekatit 12 Hospital Medical College, Addis Ababa, Ethiopia; ^2^ Department of Health Policy and Management, Faculty of Public Health, Institute of Health, Jimma University, Jimma, Ethiopia, ju.edu.et; ^3^ Department of Public Health Emergency Management, Ethiopian Public Health Institute, Addis Ababa, Ethiopia, ephi.gov.et; ^4^ Department of National Data Management and Analytics Center for Health, Ethiopian Public Health Institute, Addis Ababa, Ethiopia, ephi.gov.et

**Keywords:** associated factors, challenges, hepatitis B, opportunities, vaccination

## Abstract

**Background:**

World Health Organization reported a lacuna in receiving hepatitis B virus vaccination for a considerable proportion of healthcare workers, with coverage ranging from 18% to 39% in developing countries. In Ethiopia, full immunization coverage ranges from 5.8% to 36.9% among healthcare workers. This study is aimed at assessing the hepatitis B virus vaccination coverage, its determinants and barriers among healthcare workers in Lemi Kura Subcity, Addis Ababa, Ethiopia, 2024.

**Methods:**

A facility‐based convergent parallel mixed design was conducted in a cross‐sectional approach among 277 health workers from June 15, 2024 to July 15, 2024 in Health Centers of Lemi Kura Subcity, Addis Ababa. For the quantitative study, the retrospective samples were chosen using simple random sampling. Conversely, purposive sampling was employed for the qualitative study. Multivariable logistic regression was used to identify independent determinants of HBV vaccination. Variables with *p* value < 0.05 were considered statistically significant.

**Results:**

Hepatitis B vaccination rate was 78.3% (95% CI: 73.6, 83.0). Being female (AOR: 2.50; 95% CI: 1.21, 5.20), having more than 6 years of experience (AOR: 6.35; 95% CI: 2.33, 17.30), history of exposure to a person infected with hepatitis B (AOR: 2.88; 95% CI: 1.33, 6.21), screened for HBV (AOR: 2.72; 95% CI: 1.36, 5.41), and favorable attitude toward hepatitis B (AOR: 7.53; 95% CI: 3.11, 18.23) were statistically significant in multivariable analysis. Moreover, limited resources, misinformation, and low public awareness, workforce challenges, cost and affordability, and stigma and cultural barriers were reported challenges, and improving access, capacity building, and community engagement were reported opportunities of HBV vaccination.

**Conclusion and Recommendations:**

This study found that HBV vaccination coverage among healthcare workers in public health centers of Lemi Kura Subcity, Addis Ababa, Ethiopia, was relatively high compared with several previous reports from similar settings. Vaccination status was independently associated with sex, years of professional experience, history of occupational exposure, prior HBV screening, and attitude toward HBV vaccination.

## 1. Background

Hepatitis B virus (HBV) infection is a major global public health concern, caused by the HBV, and is associated with significant morbidity and mortality [1]. According to the World Health Organization (WHO), over 2 billion people worldwide show evidence of past or present HBV infection based on serologic testing [1, 2]. Globally, HBV infection is responsible for approximately 820,000 deaths annually, primarily due to liver cirrhosis and hepatocellular carcinoma [2]. HBV accounts for nearly 80% of liver cancer cases and is recognized as the second most significant human carcinogen after tobacco [3, 4]. The virus is transmitted parenterally through contact with contaminated body fluids, including blood, via cutaneous or mucosal exposure [5].

Healthcare workers are at increased risk of HBV infection through occupational exposures, primarily percutaneous injuries such as needlestick or sharps injuries, as well as mucosal contact with infected blood or body fluids [4]. The risk of HBV transmission following a single percutaneous exposure from an HBsAg‐positive source ranges from 6% to 30%, depending on the source′s HBeAg status [5, 6]. Mucosal exposures, particularly splash to eyes or mouth, carry lower transmission risk, estimated at 1%–6% [4, 6]. These estimates assume susceptibility in the exposed person.

WHO recommends hepatitis B vaccination for individuals at risk of HBV infection, particularly healthcare workers [6]. Consequently, it is essential to implement policies that ensure all healthcare workers are immunized and receive postexposure prophylaxis following significant exposure to patient body fluids [7, 8]. Despite this, HBV vaccination coverage among healthcare workers remains suboptimal, with only 18%–39% coverage in low‐ and middle‐income countries compared with 67%–79% in high‐income countries [9–11].

Despite the availability of an effective hepatitis B vaccine, coverage among healthcare workers in Ethiopia remains low. A national survey reported that only 5.8%–36.9% of healthcare workers were fully vaccinated, highlighting substantial gaps in occupational protection [12, 13]. Barriers to vaccination include unavailability of the vaccine, high cost, time constraints, and concerns about potential side effects [13, 14]. This low vaccination coverage represents a significant public health concern, particularly in a setting with a high burden of hepatitis B infection.

However, limited data exist on HBV vaccination status among healthcare workers in public health centers within Lemi Kura Subcity, despite increasing occurrences of the infection. This study is aimed at assessing the HBV vaccination coverage and its determinants and barriers among healthcare workers in Lemi Kura Subcity, Addis Ababa, Ethiopia. Findings will provide locally relevant evidence for policymakers and facility managers to design targeted interventions, complementing national‐level estimates and guiding operational improvements.

## 2. Methods and Materials

### 2.1. Study Design

A convergent parallel design mixed‐methods study was conducted at public health centers of Lemi Kura Subcity in Addis Ababa, the capital city of Ethiopia. Lemi Kura Subcity administration is the 11th subcity located in the east of Addis Ababa, established in 2020. Lemi Kura has an area of 7860.7 ha and a population of 382,843, organized in 10 districts, 59 clusters, and 508 blocks. There is one public hospital, nine public health centers, and three private hospitals. Public health centers in the subcity are Summit Health Center, Amoraw Health Center, Merry Health Center, Goro Health Center, Arabsa Health Center, Raey Health Center, Hidasa Health Center, Abado Health Center, and Woreda 13 Health Center. The total number of healthcare providers who are working in these public health centers is approximately 750 [15]. The study was conducted from of June 15, 2024 to of July 15, 2024.

In this approach, quantitative and qualitative data were collected for 1 month, analyzed separately, and then integrated at the interpretation stage to provide a comprehensive understanding of hepatitis B vaccination among healthcare workers.

### 2.2. Inclusion and Exclusion Criteria

All healthcare workers at public health centers at Lemi Kura Subcity were considered as source populations and all the healthcare workers at the selected public health centers were considered as study populations. Health professionals who work at the selected public health centers were included in the study. Healthcare workers were excluded from the study if they were seriously ill during data collection, unable to communicate, were on maternal leave, or had less than 6 months of experience in the facility. This criterion was applied because vaccination records and exposure experience are unlikely to be reliably established for newly hired staff. However, we acknowledge that this may introduce some selection bias and may underestimate vaccination uptake among newer staff. Those who were temporary or volunteer staff providing services without formal employment were referred to as “providing free service.” These individuals were excluded because they often lack routine access to occupational health services, including vaccination, and including them could misrepresent the typical vaccination coverage among permanent staff. In addition, those who were on leave during the study period were unable to complete the survey or interview.

### 2.3. Sample Size Determination

The sample size for the first objective was calculated using single proportion formula taking the following assumption. “*p*” is assumed to be proportion level, 45% from a study conducted in Mekelle, Northern Ethiopia is taken [16] using a higher prevalence estimate ensures a conservative sample size to achieve adequate statistical power, 95% confidence level and 5% degree of precision, *d* = margin of error (0.05), *Z* ± *α*/2 the standard normal value at 95% confidence level (1.96).

Fitting in to the formula the final sample size was *n* = (Z*α*/2)^2^ *P*( 1 − *P*)/*d* ^2^ and which gives 380. The total numbers of health professionals working at public health centers at Lemi Kura Subcity are 750. Since the total number of the source populations are less than 10,000, correction formula was used as follows: *n*_*i*_ = *n*/1 + (*n*∕*N*) = 252. In adding 10% for nonresponse rate the final sample size became 252 + 25 = 277. For the qualitative component, 16 individuals participated in the focus group discussions (FGDs), and 8 in‐depth interviews (IDIs) were conducted in two groups and the principle of saturation was adhered [17].

Among the nine public health centers found in the subcity, five health centers were selected by using a simple random sampling. These health centers are Woreda 13 Health Center, Goro Health Center, Arabsa Health Center, Abado Health Center, and Summit Health Center. The lists of the health professionals were collected from the human resource management of each subcity to use it as a sampling frame. The sample size was allocated for each selected health center proportionally based on the number of health professionals. Finally, simple random sampling was applied at each health center. Three repeated attempts were made for the study participants who were not available during the data collection period. For the qualitative part of the study, a purposive sampling technique was employed to select participants [17].

### 2.4. Study Variables

The dependent variable was hepatitis B vaccination status and the independent variables were sociodemographic factors (age, sex, educational status, marital status, monthly income, and profession), knowledge, attitude, and health facility and work‐related factors (working unit, length of experience, direct patient contact, training, vaccine availability, and occupational exposure).

Good knowledge: If a respondent scored ≥ 70 correct responses on the knowledge assessment questions, the respondent has good knowledge. Poor knowledge: If a respondent scored below 70 correct responses on the knowledge assessment questions, the respondent has poor knowledge. Attitude toward HBV vaccination was assessed using a structured questionnaire adapted from previously published studies conducted among healthcare workers assessed using 11 questions [9, 11, 13, 14]. Its scale consisted of Likert‐scale items designed to measure perceptions regarding vaccine effectiveness, safety, necessity, and personal susceptibility to infection. Positive attitude: If respondents scored ≥ 70 correct responses on the attitude assessment items, they have positive attitude; negative attitude: If respondents scored below 70 correct responses on the attitude assessment items, they have negative attitude.

Healthcare workers are healthcare personnel who are directly or indirectly exposed to patients, blood, body fluids or other potentially infectious materials during the course of their work, which include physicians, midwives, nurses, health officers, anesthetists, dentists, and laboratory technicians and supportive staff [16]. Vaccination status is the status of being vaccinated with HBV vaccine depending on the number of doses received categorized into unvaccinated, partially vaccinated, and fully vaccinated [16], unvaccinated HCPs are HCPs who did not receive any doses of HBV vaccine [16], partially vaccinated HCPs are HCPs who received only one or two doses of HBV vaccine [16], and fully vaccinated HCPs are HCPs who received a full course (three doses) of HBV vaccine [16].

### 2.5. Data Collection

The questionnaire was initially developed in English and subsequently translated into Amharic, the local language, before being back‐translated to English to ensure consistency and accuracy. The data collection tool was adapted from multiple studies conducted on similar topics [16–20]. It consisted of four sections: sociodemographic factors, attitudes, knowledge, and facility‐related factors. Data collection was carried out by four trained BSc nurses under the supervision of two MSc‐qualified nurses, utilizing a pretested structured questionnaire. Quantitative data were collected using self‐administered structured questionnaires distributed to selected healthcare workers during working hours. Questionnaires were completed on‐site and returned immediately to the data collectors. Data collectors reviewed each questionnaire for completeness before accepting submission. If any items were incomplete, participants were politely asked to complete the missing responses. Both IDIs and FGDs were employed to gather rich qualitative data.

The qualitative component included 20 IDIs and three FGDs conducted among healthcare workers in Lemi Kura Subcity. IDIs were conducted in a private and quiet setting to ensure confidentiality and encourage open expression. Each FGD was audio‐recorded with participants′ informed consent and held in neutral and accessible venues, with each session comprising eight participants and lasting 90–120 min. A trained facilitator led the discussions using a semistructured guide, whereas a note‐taker documented key points, nonverbal cues, and group dynamics. For both IDIs and FGDs, field notes were taken to capture contextual details and interviewer/facilitator reflections. The guides were designed to explore participants′ experiences, perceptions, and challenges, with flexibility to probe emerging issues while ensuring coverage of the study objectives [17]. Data collection continued until thematic saturation was achieved, defined as the point at which no new themes or substantive insights emerged from subsequent interviews.

### 2.6. Quality Control Measures

A 1‐day intensive training session was conducted by the principal investigator for both data collectors and supervisors. The training covered the study′s objectives, procedures, data collection methods, and guidance on managing the data collection process. A pretest of the questionnaire was conducted with 5% of the study participants at Hidase Health Center 1 week prior to the data collection and its alpha score was 78%. Adjustments were made to the questionnaire based on the pretest findings to ensure clarity and improve response accuracy. Collected data were reviewed and checked daily for completeness and relevance before being entered. Finally, data were entered into EpiData Version 4.6.

Qualitative data were translated, transcribed, and analyzed using established qualitative analysis techniques. The findings from the qualitative component were appropriately triangulated with the quantitative results. The transcription process for FGDs and IDIs was rigorous. Skilled and certified professionals handled the transcription and translation tasks. To ensure accuracy, two independent transcribers reviewed the audio recordings and transcribed participants′ responses verbatim. Any discrepancies between the transcriptions and audio recordings were carefully cross‐checked through member validation, and differences were resolved. The finalized transcriptions were subsequently translated into English [17].

To ensure the trustworthiness of the study, essential criteria, including credibility, dependability, confirmability, and transferability, were thoroughly addressed. The meticulous transcription and translation process were designed to uphold the accuracy, consistency, and reliability of the qualitative data, thereby strengthening the credibility and validity of the study′s findings.

### 2.7. Data Analysis

Data entry was undertaken using EpiData Version 4.6 and analysis was conducted using SPSS 27 for Windows. Following data entry, data cleaning was conducted to avoid any missing values, outliers, and other inconsistencies before analysis. Descriptive studies like measures of central tendency and measures of dispersion for continuous data, and frequency count and proportion for categorical data were used to summarize descriptive data. Associations between categorical variables are now explicitly assessed using chi‐squared tests.

Bivariate logistic regression was used to select candidate variables for multivariable logistic regression. Multivariable logistic regression was used to identify independent factors associated with HBV vaccination to control confounders. Adjusted odds ratio (AOR) and confidence interval (CI) were, respectively, used to measure the association between HBV vaccination and their statistical significance in the final model. Hosmer and Lemeshow test was used to check model fitness. Multicollinearity was assessed using variance inflation factor (VIF) and tolerance statistics. All VIF values were below 3, indicating the absence of significant multicollinearity. Variables with *p* ≤ 0.25 in bivariable analysis were entered into multivariable logistic regression. Backward likelihood ratio method was used while retaining epidemiologically important variables.

Potential interaction effects between experience and training, experience and screening, and training and attitude were assessed; none were statistically significant. Finally, AOR with 95% CI was used to identify factors associated with HBV vaccination and statistical significant variables were declared at a *p* value < 0.05.

The qualitative data were first transcribed verbatim and translated into English to ensure accuracy and consistency. The translated transcripts were then imported into Atlas.ti software for systematic organization and analysis. A thematic analysis approach was employed, beginning with repeated reading of the transcripts to achieve familiarity with the data. The categories were refined into overarching themes that captured the key patterns and meanings within the data. The themes were interpreted in relation to the study objectives and supported by direct quotes from participants to enhance credibility and illustrate the findings. The trustworthiness of the data was ensured. This methodological integration allowed for a comprehensive understanding of the research topic, enriching the overall analysis and interpretation of the study results.

## 3. Results

### 3.1. Sociodemographic Characteristics

This study was conducted with a total of 277 study participants, which yields a response rate of 100%. The age of participants ranged from 23 to 45 years, with a median age of 32 (IQR: 6) years. Nearly one‐quarter (24.5%) of respondents were aged 23–25 years old, whereas 14.4% were aged 25–29 years old. The majority of the study participants were female (198, or 71.5%), and 133 (48.0%) held a first degree (Table 1).

**Table 1:** Sociodemographic characteristics of healthcare workers at public health centers in Lemi Kura Subcity, Addis Ababa, Ethiopia; 2024.

Variables	Category	Frequency (*n*)	Percentage (%)
Age (in years)	20–24	68	24.5%
	25–29	40	14.4%
	30–34	43	15.5%
	35–39	42	15.2%
	More than 39	84	30.3%
Sex	Male	79	28.5%
	Female	198	71.5%
Level of education	Diploma	98	35.4%
	First degree	133	48.0%
	Second degree and above	46	16.6%
Marital status	Currently not married	201	72.6%
	Currently married	76	27.4%
Estimated monthly income (in ETB)	Less than 5000	32	11.6%
	5000–10,000	196	70.8%
	More than 10,000	49	17.7%

### 3.2. Profession, Work and Related Characteristics

More than half of the study participants (152, or 54.9%) were nurses and 53 (19.1%) were medical laboratory technicians. About one‐fourth of the participants (69, or 24.9%) worked in the inpatient department, whereas 84 (30.3%) worked in the outpatient department. In terms of work experience, 49 (17.7%) had worked for less than 2 years, and 81 (29.2%) had more than 6 years of work experience. Majority of the study participants (221, 79.8%) took infection prevention training (Table 2).

**Table 2:** Profession and work‐related characteristics of healthcare workers at public health centers in Lemi Kura Subcity, Addis Ababa, Ethiopia; 2024.

Variables	Category	Frequency (*n*)	Percentage (%)
Profession	Nurse	152	54.9%
	Public health officers	31	11.2%
	Medical laboratory technician	53	19.1%
	Midwife	31	11.2%
	Others	10	3.6%
Working department	Emergency	37	13.4%
	Labor and delivery	34	12.3%
	Laboratory	53	19.1%
	Inpatient	69	24.9%
	Outpatient	84	30.3%
Years of experience	Less than two	49	17.7%
	2–4	69	24.9%
	4–6	78	28.2%
	More than six	81	29.2%
Took infection prevention training	Yes	221	79.8%
	No	56	20.2%

### 3.3. Hepatitis B Exposure, Testing, and Related Characteristics

Among the study participants, 131 (47.3%) had encountered exposure to someone diagnosed with hepatitis B. Of those exposed, 50 (38.2%) had encounters within the past year, 53 (40.5%) within the past 2–3 years, and 28 (21.4%) more than 3 years ago. In terms of actions taken after exposure, 69 (52.7%) rinsed the area with water, whereas 62 (47.3%) received postexposure prophylaxis. Of those healthcare and subsidiary workers who had been screened for HBV, 176 individuals (63.5%) were screened at least once in their lifetime. Among these, 76 (43.2%) had been screened within the past year, 60 (34.1%) within the past 2–3 years, and 40 (22.7%) more than 3 years ago. Among the respondents who were not screened for hepatitis B, the reasons were as follows: 5 (5.0%) cited cost, 21 (20.8%) cited low perceived need, 60 (59.4%) feared a positive result, and 15 (14.9%) were too busy with their workload.

### 3.4. Knowledge on Hepatitis B

The proportion of healthcare workers who have good knowledge on hepatitis B was 68.6% (95% CI: 63.2, 74.0). Among the study participants, 48 (17.7%) mentioned that hepatitis B is caused by bacterial infection and 265 (95.7%) stated Hepatitis B can be transmitted through contact with infected blood (Table 3).

**Table 3:** Knowledge on hepatitis B vaccination among healthcare workers at public health centers in Lemi Kura Subcity, Addis Ababa, Ethiopia; 2024.

Item	Yes (*n*[%])	No (*n*[%])
Hepatitis B is caused by bacterial infection.	48 (17.7%)	228 (82.3%)
Hepatitis B can be transmitted through contact with infected blood.	265 (95.7%)	12 (4.3%)
There is a vaccine available to prevent Hepatitis B infection.	260 (93.9%)	17 (6.1%)
Hepatitis B became chronic infection.	272 (98.2%)	5 (1.8%)
Hepatitis B infection is always symptomatic in infected individuals.	38 (13.7%)	239 (86.3%)
Hepatitis B leads to liver cancer if left untreated.	276 (99.6%)	1 (0.4%)

A study participant from an IDI stated that “….HBV is a viral infection that affects the liver and can cause both acute and chronic health problems. It′s primarily transmitted through contact with infected blood and body fluids. For healthcare workers, the risk of exposure is particularly high because of the nature of our work handling blood, performing surgeries, and treating patients with open wounds. The virus can be transmitted through needle sticks, cuts, or contact with contaminated fluids. If a healthcare worker gets infected, there′s a risk of developing serious liver conditions, like cirrhosis or liver cancer.” Another study participant from an IDI stated that “…I′ve heard that the hepatitis B vaccine is highly effective in preventing the infection, but I know there are still many healthcare workers who remain unvaccinated, which is concerning, especially since we are at a greater risk due to constant patient interaction.”

### 3.5. Attitude Toward Hepatitis B Vaccination

The proportion of healthcare workers who have favorable attitude toward hepatitis B vaccination was 86.6% (95% CI: 82.3, 90.3). Among the study participants, 109 (39.4%) agree that receiving the hepatitis B vaccine is essential for safety as a healthcare worker and 124 (44.8%) hepatitis B vaccine is effective in preventing infection (Table 4).

**Table 4:** Attitude toward hepatitis B vaccination among healthcare workers at public health centers in Lemi Kura Subcity, Addis Ababa, Ethiopia; 2024.

Item	Strongly disagree	Disagree	No opinion	Agree	Strongly agree
Receiving the hepatitis B vaccine is essential for safety as a healthcare worker.	10 (3.6%)	12 (4.3%)	12 (4.3%)	109 (39.4%)	134 (48.4%)
Hepatitis B vaccine is effective in preventing infection.	7 (2.5%)	12 (4.3%)	10 (3.6%)	124 (44.8%)	124 (44.8%)
It is important for all healthcare workers to be vaccinated against hepatitis B.	9 (3.2%)	19 (6.9%)	13 (4.7%)	124 (44.8%)	112 (40.4%)
The benefits of the hepatitis B vaccine outweigh any potential risks.	6 (2.2%)	8 (2.9%)	5 (1.8%)	126 (45.5%)	132 (47.7%)
Healthcare institutions should mandate hepatitis B vaccination for their employees.	0 (0.0%)	0 (0.0%)	34 (12.3%)	184 (66.4%)	59 (21.3%)
Hepatitis B vaccination should be a priority in healthcare settings.	6 (2.2%)	5 (1.8%)	9 (3.2%)	165 (59.6%)	92 (33.2%)
Hepatitis B vaccination is a crucial part of infection control in healthcare settings.	8 (2.9%)	7 (2.5%)	11 (4.0%)	154 (55.6%)	97 (35.0%)
Getting vaccinated against hepatitis B demonstrates professionalism as a healthcare worker.	4 (1.4%)	9 (3.2%)	8 (2.9%)	130 (46.9%)	126 (45.5%)
Healthcare facilities should support and encourage vaccination against hepatitis B.	9 (3.2%)	11 (4.0%)	9 (3.2%)	136 (49.1%)	112 (40.4%)
Hepatitis B vaccine is necessary even if good hygiene and safety protocols practiced.	5 (1.8%)	8 (2.9%)	6 (2.2%)	147 (53.1%)	111 (40.1%)
Regular updates and reminders about hepatitis B vaccination are important for healthcare workers.	10 (3.6%)	12 (4.3%)	19 (6.9%)	154 (55.6%)	82 (29.6%)

### 3.6. Hepatitis B Vaccination Status

**Figure 1:**
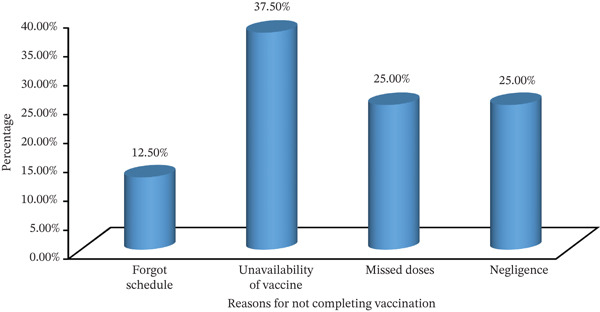
Reasons for not completing the vaccine among healthcare workers at public health centers in Lemi Kura Subcity, Addis Ababa, Ethiopia; 2024.

Among the respondents, 217 (78.3%) completed hepatitis B vaccine. Therefore, the proportion of healthcare workers who were fully vaccinated was 78.3% (95% CI: 73.6, 83.0). In addition, 20 (7.20%) were unvaccinated, and 40 (14.5%) were incomplete. The most common reasons for not getting vaccinated were the unavailability of the vaccine through government channels (7, 35.0%), fear of side effects (6, 30.0%), negligence (4, 20.0%), and fear of needles (3, 15.0%). Among those who did not complete the full vaccination series, 15(37.5%) cited the unavailability of the vaccine at the time of exposure as the reason (Figure 1).

## 4. Challenges and Opportunities

### 4.1. Challenges

#### 4.1.1. Limited Resources

Study participants from FGD stated as “……….we don′t have a consistent supply of hepatitis vaccines. Sometimes, we have to ask patients to come back in a week or more, which frustrates them.” Another study participant from an IDI mentioned that, “….. Our facility lacks enough storage space for vaccines, and we have to rely on other centers to store them properly.” Another study participant stated that “Sometimes the health center requests the vaccine, but it does not arrive on time. Even if staffs are willing, they cannot complete the doses.” *(Health officer, 10* years*′ experience, IDI).* Some participants also linked stock‐outs to cold chain and logistics issues. “They say the vaccine must be kept properly, but our facility sometimes has power interruptions. That affects storage and supply.” (Laboratory professional, 5 years′ experience, IDI).

#### 4.1.2. Misinformation and Low Public Awareness

A study participant from an IDI reported that, “……… most people in this community don′t know how serious hepatitis is, and they think the vaccine is unnecessary unless they are already sick.” Another study participant from an IDI mentioned that, “……… there′s a widespread belief that the vaccine has side effects like infertility. It′s very hard to convince people otherwise.”

#### 4.1.3. Workforce Challenges

Study participants from FGD reported that “…….we are short‐staffed, and this affects the quality of services we provide. Vaccination is just one of many things we have to do daily.” Another study participant from an IDI mentioned that “…….. the workload is high, but there′s no recognition or support for healthcare workers handling vaccination programs.”

#### 4.1.4. Cost and Affordability

Study participants from FGD reported that “…… while the vaccine is free in public facilities, some patients say they can′t afford the transport fare to come here.” Similarly, they mentioned that “…. the cost of hepatitis testing before vaccination is another barrier for many patients in this area.”

#### 4.1.5. Stigma and Cultural Barriers

A study participant from an IDI stated that “……. some patients think getting vaccinated means admitting you have hepatitis, which discourages them from seeking the service.” Another study participant from an IDI stated that “….young adults, especially, are hesitant because they fear being judged by their peers or family members.”

### 4.2. Opportunities

#### 4.2.1. Improving Access

Study participants from FGD mentioned that “… if we had mobile vaccination services in Lemi Kura, we could reach more people, especially those in informal settlements.” Moreover, they stated that “…. integrating hepatitis vaccination with other programs like antenatal care has worked well in other areas, and we should try it here too.”

#### 4.2.2. Capacity Building

A study participant from an IDI stated that “…. regular refresher training would help us communicate better with patients and handle their concerns about the vaccine.” Another study participant from an IDI mentioned that “…. we need more updates on vaccination protocols and strategies to deal with vaccine hesitancy in this community.”

#### 4.2.3. Community Engagement

Study participants from FGD stated that “…. engaging local community leaders and religious figures would be a game‐changer. People trust them and listen to their advice.” In addition, a study participant from an IDI reported that “…we′ve seen that community dialogues can help reduce resistance. It’s time we held more of those.”

#### 4.2.4. Determinants of Hepatitis B Vaccination Status

Sex, years of experience, HBV exposure, screening for HBV, knowledge on HBV, and attitude were candidate variables on bivariate analysis. Among them, sex, years of experience, HBV exposure, screening for HBV, and attitude were statistically significant in the multivariable logistic regression model.

Female healthcare workers were 2.5 times more likely to be vaccinated against hepatitis B compared with their male counterparts (AOR: 2.50; 95% CI: 1.21, 5.20). The odds of being vaccinated against hepatitis B were 6.35 times higher among healthcare workers with more than 6 years of experience compared with those with less than 2 years (AOR: 6.35; 95% CI: 2.33, 17.30). Similarly, healthcare workers with 4–6 years of experience had 6.26 times higher odds of vaccination (AOR: 6.26; 95% CI: 2.38, 16.46) compared with those with less than 2 years of experience. Moreover, those with 2–4 years of experience had nearly five times higher odds of being vaccinated (AOR: 5.29; 95% CI: 1.99, 14.05) compared with the same reference group.

Healthcare workers who have a history of exposure to a person infected with hepatitis B had 2.88 times higher odds of being vaccinated compared with those who did not encounter exposure (AOR: 2.88; 95% CI: 1.33, 6.21). The odds of being vaccinated against hepatitis B were 2.72 times higher among healthcare workers who were screened compared with those who were not screened (AOR: 2.72; 95% CI: 1.36, 5.41). Additionally, healthcare workers with a favorable attitude toward hepatitis B vaccination were 7.53 times more likely to be vaccinated compared with those with an unfavorable attitude (AOR: 7.53; 95% CI: 3.11, 18.23) (Table 5).

**Table 5:** Factors associated with hepatitis B vaccination status among healthcare workers at public health centers in Lemi Kura Subcity, Addis Ababa, Ethiopia; 2024.

Variables	Category	HBV vaccinated		*X* ^2^ (*p* value)	COR (95% CI)	AOR (95% CI)
		Yes	No			
Sex	Male	54	25	6.494 (0.010)	1	1
	Female	163	35		2.16 (1.19, 3.92) ^∗^	2.50 (1.21, 5.20) ^∗∗^
Experience (in years)	Below two	27	22	21.64 (0.0001)	1	1
	2–4	53	16		2.69 (1.22, 5.97) ^∗^	5.29 (1.99, 14.05) ^∗∗^
	4–6	66	12		4.48 (1.95, 10.32) ^∗^	6.26 (2.38, 16.46) ^∗∗^
	More than 6	71	10		5.79 (2.43, 13.79) ^∗^	6.35 (2.33, 17.30) ^∗∗^
Encountered exposure with infected person	Yes	115	16	13.07 (0.0001)	3.10 (1.65, 5.83) ^∗^	2.88 (1.33, 6.21) ^∗∗^
	No	102	44		1	1
Ever screened for HBV	Yes	151	25	15.81 (0.0001)	3.20 (1.78, 5.77) ^∗^	2.72 (1.36, 5.41) ^∗∗^
	No	66	35		1	1
Knowledge on HBV	Poor	57	30	12.29 (0.001)	1	1
	Good	160	30		2.81 (1.56, 5.06) ^∗^	1.76 (0.86, 3.59)
Attitude toward HBV vaccination	Unfavorable	19	18	18.33 (0.0001)	1	1
	Favorable	198	42		4.47 (2.16, 9.23) ^∗^	7.T (3.11, 18.23) ^∗∗^

^∗^indicates variables with *p* value < 0.25 in bivariate analysis.

^∗∗^indicates variables with *p* value < 0.05 in multivariable analysis.

A study participant from an IDI stated that “*…* I′ve seen how easily the virus can spread, especially in situations where there′s a lot of exposure to blood and bodily fluids, and I trust the vaccine′s effectiveness—being protected from such a serious disease gave me peace of mind.” Another study participant from an IDI reported that “…. my workplace encouraged vaccination and provided the vaccine free of charge, which made it easier to access. I also spoke to colleagues who had been vaccinated and heard about their positive experiences.” In addition, a study participant from FGD mentioned that “….the fact that I care about my own health and the health of my patients was a major influence. I didn′t want to risk contracting or spreading hepatitis B, so getting vaccinated seemed like the responsible choice.”

#### 4.2.5. Quantitative Integration With Qualitative Finding

Female healthcare workers described higher perceived vulnerability and stronger preventive attitudes. “As a woman, especially if I plan to have children, I am more concerned about infections like hepatitis.” *(Nurse, 6* years*′ experience, IDI) and another participant responded* “Female staffs usually take vaccination more seriously than male staff.”*(Health officer, 9* years*′ experience, IDI-12).* These narratives align with the quantitative finding that female sex was significantly associated with higher vaccination uptake.

Many participants indicated that HBV vaccination was not systematically offered during recruitment or orientation. “When I was employed, nobody asked about my hepatitis B vaccination status.” (Midwife, 4 years′ experience, IDI) and another participant stated that “There is no formal policy that new staff must be vaccinated before starting work.” (Environmental health officer, 5 years′ experience, IDI). This institutional gap helps explain the association between years of experience and vaccination status, as longer‐serving staff were more likely to have accessed vaccination opportunities over time.

Participants emphasized that prevaccination screening costs discouraged vaccine uptake. “The vaccine itself may be available sometimes, but the screening test costs money. That is why many delay.” *(Pharmacist, 6* years*′ experience, IDI).* This supports the quantitative finding that prior HBV screening was significantly associated with vaccination uptake.

Participants reported absence of clear institutional vaccination policy and weak enforcement mechanisms. For example, “There is no written rule that healthcare workers must complete hepatitis B vaccination.” *(Medical director, 9* years*′ experience, IDI),* “If there was a strict policy from the subcity health office, everyone would be vaccinated.” *(Laboratory professional, 7* years*′ experience, FGD). Moreover, s*everal participants linked this to workforce protection gaps as “We handle blood daily, but occupational safety is not prioritized.”*(Nurse, 11* years*′ experience, FGD).* This theme explains the strong association between positive attitude and vaccination status observed in the quantitative findings.

## 5. Discussion

The findings of this study offer important insights into hepatitis B vaccination among healthcare workers in an urban Ethiopian setting. Unlike national estimates, our facility‐level data highlight both successes and gaps in vaccination coverage, providing a nuanced understanding of determinants and barriers. The study is novel in documenting context‐specific challenges and opportunities within Lemi Kura Subcity, thereby filling a critical evidence gap. These findings can directly inform occupational health policies, guide targeted vaccination programs, and serve as a model for scaling interventions to similar urban settings in Ethiopia. Moreover, understanding local facilitators of vaccination may help predict vaccination uptake trends and strengthen future policy decisions aimed at achieving universal healthcare worker immunization.

In this study, 78.3% (95% CI: 73.6, 83.0) of healthcare workers reported completion of the full three‐dose hepatitis B vaccination schedule. This study finding was consistent with the studies finding conducted in Kuwait (74.7%) [21]. It was higher than the WHO reported estimates for healthcare workers in low‐ and middle‐income countries (18%–39%) [15, 17, 18]. This discrepancy may reflect contextual differences, as Addis Ababa is an urban setting with relatively improved healthcare infrastructure, better vaccine availability, and greater institutional enforcement of occupational health policies compared with many other resource‐limited settings.

Consistently, this study showcased higher vaccination coverage than the studies conducted in Georgia (12%) [22], Pakistan (53%) [23], Iraq (45%) [24], India (35%) [25], Nigeria (36.25%) [26], Bulle Hora Woreda, Southern Oromia (1.2%) [27], St. Paul′s and Zewditu hospital, Addis Ababa (3.5%) [28], Harar (4.7%) [29], and Bahir Dar City Administration, North West Ethiopia (5.4%) [17]. These differences might be related to sociodemographic characteristics, cultural attitudes toward vaccination, and healthcare practices.

The sex of the healthcare provider was found to be a factor associated with hepatitis B vaccination status. Consistent with the studies conducted in India [21], Wolayta Soddo Hospital [30], and Addis Ababa [18], female healthcare workers were 2.5 times more likely to be vaccinated against hepatitis B compared with their male counterparts. This may reflect that female healthcare workers tend to exhibit more proactive health‐seeking behaviors and are more likely to adhere to vaccination recommendations and health guidelines compared with male healthcare workers [5, 11]. Additionally, women may perceive a higher risk of contracting hepatitis B, leading them to prioritize vaccination more [11]. Furthermore, female healthcare workers might also be more compliant with institutional vaccination policies. In the study setting, female healthcare workers are disproportionately represented in nursing and midwifery roles, which involve greater patient‐facing responsibilities and participation in maternal and child health programs. Exposure to antenatal care (ANC) services where vaccination practices are routinely emphasized may contribute to a stronger vaccination culture among female staff. Therefore, the observed association may partly reflect occupational role distribution and service‐unit exposure rather than sex alone.

Increased years of work experience encourage hepatitis B vaccination. The odds of being vaccinated against hepatitis B were 6.35 times higher among healthcare workers with more than 6 years of experience compared with those with less than 2 years. Similarly, healthcare workers with 4–6 years of experience had 6.26 times higher odds of vaccination compared with those with less than 2 years of experience. Moreover, those with 2–4 years of experience had nearly five times higher odds of being vaccinated compared with the same reference group. This study finding is consistent with the studies conducted in Shashemene Zonal Town [19], Wolaita Soddo [30] and public hospitals in Mekelle, Northern Ethiopia [16]. Years of professional experience likely reflect cumulative exposure to occupational risk, institutional vaccination campaigns, and infection prevention training, which may explain the strong association with vaccination uptake.

This could be explained by experienced healthcare workers being more familiar with the occupational risks associated with hepatitis B and thus recognizing the importance of vaccination [16]. Over time, they receive more training and education on infection control and the benefits of vaccination, which can lead to higher vaccination rates [19]. Additionally, long‐serving employees may reflect the implementation and strengthening of institutional vaccination policies, making them more likely to be vaccinated [19]. Workers with more experience are also more likely to have encountered exposure incidents, encouraging them to get vaccinated as a precaution [16, 19]. Moreover, they often have better access to occupational health services and vaccination programs, and have had more opportunities to receive the vaccine through workplace campaigns or health initiatives [11]. Experienced workers may also be more focused on their long‐term health and job security, motivating them to take preventive measures such as vaccination [31, 32].

Encountered exposure with an infected person was identified as a factor associated with hepatitis B vaccination. Healthcare workers who have a history of exposure to a person infected with hepatitis B had 2.88 times higher odds of being vaccinated compared with those who did not encounter exposure. This study finding was in line with the study finding conducted in public hospitals in Mekelle, Northern Ethiopia [16]. This may be because healthcare workers who have had direct exposure to hepatitis B–infected individuals tend to be more conscious of the risks associated with the virus, prompting them to prioritize vaccination as a preventive measure [16]. Additionally, exposure incidents often activate institutional protocols that include vaccination or postexposure prophylaxis, encouraging those affected to get vaccinated to prevent future infections [30, 33]. Furthermore, workers who have experienced exposure are more inclined to pursue preventive health measures, including vaccination, as part of their strategy to safeguard themselves against infection [15, 16].

Screening for HBV was found to be a factor associated with hepatitis B vaccination. In line with the study findings conducted in Akaki Kality Subcity, Addis Ababa [33], the odds of being vaccinated against hepatitis B were 2.72 times higher among healthcare workers who were screened compared with those who were not screened. This could be because screening often raises awareness about Hepatitis B and the need for vaccination, making those who are screened more likely to get vaccinated [26]. Additionally, screening identifies individuals who are not immune to hepatitis B, and those found to be at risk are more likely to be advised to get vaccinated [30, 33]. Furthermore, people who undergo screening may receive follow‐up care that includes vaccination as part of their preventive or treatment plan [15, 16, 20, 34]. HBV screening was significantly associated with vaccination status. However, due to the cross‐sectional design, the direction of this relationship cannot be determined. Although screening may facilitate vaccination uptake by identifying susceptibility, it is also plausible that healthcare workers who are vaccinated are more likely to undergo screening as part of occupational health procedures or bundled vaccination services. Therefore, reverse causality and shared underlying health‐seeking behavior cannot be excluded.

A favorable attitude promotes hepatitis B vaccination among healthcare workers. Healthcare workers with a favorable attitude toward hepatitis B vaccination were 7.53 times more likely to be vaccinated compared with those with an unfavorable attitude. This study finding was consistent with the study conducted in Wolaita Soddo [30]. Healthcare workers with a positive attitude toward hepatitis B vaccination are more likely to be motivated to get vaccinated and adhere to vaccination recommendations [21, 35]. A favorable attitude often involves recognizing the benefits of vaccination, such as personal protection and preventing the spread of hepatitis B, which encourages vaccination [25]. Although positive attitude was significantly associated with vaccination status, reverse causality cannot be excluded. Healthcare workers who received vaccination may subsequently develop more favorable perceptions due to an increased sense of protection, institutional messaging, or positive vaccination experiences. Therefore, the observed association may reflect bidirectional influence rather than a unidirectional effect of attitude on vaccination uptake.

The cross‐sectional design limits the ability to establish temporal sequence or causality between predictors and vaccination status. Additionally, the study did not include hepatitis B testing. Moreover, the study participants′ vaccination status was ascertained using a self‐report; it may introduce a recall bias and social desirability bias. As hepatitis B vaccination is considered an occupational health responsibility, participants may have over‐reported vaccination uptake or positive attitudes to align with perceived professional expectations. Consistently, knowledge and attitude were categorized using mean score cutoffs. This approach may lead to misclassification and loss of variability, potentially attenuating or inflating associations. In addition, healthcare workers with less than 6 months of employment were excluded. This may have underestimated or overestimated vaccination coverage, as newly recruited staff may differ systematically in vaccination status or exposure to institutional programs. Moreover, the wide CI observed for attitude may reflect limited statistical power and should be interpreted cautiously.

## 6. Conclusion

This study found that three doses of HBV vaccination and the coverage among healthcare workers in public health centers of Lemi Kura Subcity, Addis Ababa, Ethiopia, were relatively high compared with several previous reports from similar settings. Vaccination status was independently associated with sex, years of professional experience, history of occupational exposure, prior HBV screening, and attitude toward HBV vaccination. However, given the cross‐sectional approach, these associations should not be interpreted as generic. Qualitative findings further highlighted institutional challenges, including vaccine stock‐outs, screening cost barriers, and absence of structured workplace vaccination policies. Therefore, strengthening occupational health policies is required to ensure systematic HBV vaccination coverage among all healthcare workers and strengthening vaccine supply chain management is indispensable to prevent stock‐outs.

**Nomenclature**
AOR: adjusted odds ratio
CI: confidence interval
COR: crude odds ratio
HBeAg: hepatitis B e‐antigen
HBV: hepatitis B virus
HCW: healthcare workers
HC: health center
HIV: human immune virus
LKSC: Lemi Kura Subcity
WHO: World Health Organization

## Author Contributions

Samuel Dessu Sifer: conceptualization, methodology, investigation, data curation, formal analysis, writing—original draft, and writing—review and editing. Nebyu Lakew Tefera: methodology, validation, software, and writing—review and editing. Abenezer Zenebe Kebede: methodology, validation, software, and writing—review and editing. Melkamu Abte: project administration, resources, validation, and writing—review and editing. Mulugeta Geremew: project administration, resources, validation, visualization, and writing—review and editing. All authors were involved in visualization, writing the original draft, review and editing of the manuscript.

## Funding

No funding was received for this manuscript.

## Ethics Statement

Ethical approval was received from Yanet College Institutional Review Board with a letter written using a reference number YC/IRB/062/2024. The written letter was submitted to each health center for permission and to the mothers to secure their consent. Each participant was given their written consent after clearly presenting the study′s objectives, benefits, and risks, as well as their right to decide whether or not to participate in the study. For the sake of confidentiality, their names were omitted. The respondents were interviewed in a secured place. The study was conducted in compliance with the Helsinki Declaration.

## Consent

The authors have nothing to report.

## Conflicts of Interest

The authors declare no conflicts of interest.

## Data Availability

Data available in article Supporting Information.
